# Digitizing "The NBS Tables of Chemical Thermodynamic Properties: Selected Values for Inorganic and C1 and C2 Organic Substances in SI Units"^1^1Certain software products are identified in this paper in order to specify the procedure adequately. Such identification is not intended to imply recommendation or endorsement by NIST, nor is it intended to imply that the products identified are necessarily the best available for the purpose.


**DOI:** 10.6028/jres.125.007

**Published:** 2020-02-04

**Authors:** Janiel J. Reed

**Affiliations:** 1 National Institute of Standards and Technology,Gaithersburg, MD 20899, USA

**Keywords:** chemical thermodynamics, enthalpy, entropy, evaluated data, Gibbs energy, inorganic chemistry, NBS Tables, thermochemistry

## 1 Summary

The NBS Tables of Chemical Thermodynamic Properties [1] is a collection of thermodynamic properties, published in book form, consisting of 103 tables with 14 330 critically evaluated species. The tables were originally published as a series of NBS^2^2 National Bureau of Standards, now NIST, National Institute of Standards and Technology. Technical Notes [2]. As a result of this work, the data is now available in a more accessible spreadsheet format. Enthalpy of formation, Δ_f_H°, Gibbs energy of formation, Δ_f_G°, entropy, S°, heat capacity at constant pressure, C_p_°, all at 298.15 K, and the enthalpy difference, [H°(298) - H°(0)] are provided where known. Within this collection of data, there are no values given for transuranic elements, Np to Lr (Tables 77-87).

## **Table 2.** Data Specifications

**Table:** 

**NIST Operating Unit(s)**	Material Measurement Laboratory, Chemical Sciences Division, Chemical Informatics Research Group
**Format**	CSV, Excel
**Accessibility**	All datasets submitted to *Journal of Research of NIST* are publicly available.
**License**	https://www.nist.gov/director/licensing

## 3 Methods

**Fig. 1:**
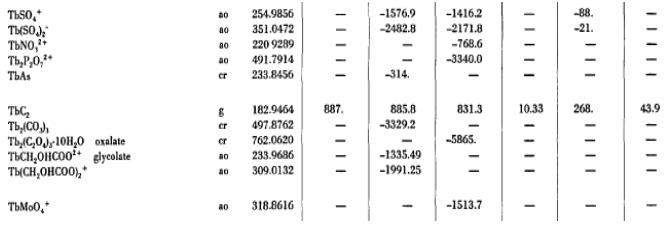
A section of the Tables copied from the original PDF file.

**Fig. 2:**
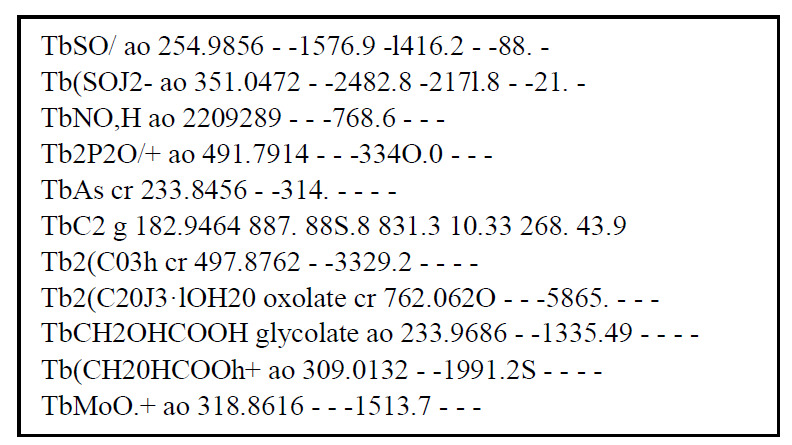
The same section of the Tables in Fig. 1, shown here as it appears when copied from the PDF file into an MS Word file.

The NBS Tables of Chemical Thermodynamic Properties [1] book was digitized (optical character recognition - OCR) into a portable document format (PDF) file. The tables within the PDF file were copied (Fig. 1) and pasted into Microsoft Word (Fig. 2). Within Word, column alignments and chemical formulas were fixed using the PDF as a guide. From Word, the data were then copied into Excel and printed. Using the original book as a guide, all tables were manually edited and corrected. After editing, the contents of the spreadsheet were checked using a Python script. State identifiers were checked for consistency and numeric values were checked for format and range. An erratum [4] was used to include corrected values in the data which is indicated with an asterisk. Due to the small amount of values from the erratum [4], they were checked manually.

Figure 1 below shows the digitized results of a section of the Tables for terbium and Fig. 2 shows the same content after it has been copied into Microsoft Word.

The methods used to compile and evaluate the data are provided in chapters two through six of Ref. [1]. Table 1 describes each column in the Excel file.

**Table 1:** Description of each column in the Excel file.

Heading	Description
Formula	Written in conventional manner
Solvent	Physical state that is normal for the indicated solvent at 298.15 K (almost always liquid). May be a mixture
Name	Name of given formula
State Description	Additional information about the state of the species
State	Physical state of each substance is indicated as crystalline solid (cr), liquid (l), vitreous or glassy (vit), amorphous (am), or gaseous(g)
Molar Mass (g mol^-1^)	Molar mass in gram per mole of substance
0 K Δ_f_H° (kJ mol^-1^)	Enthalpy of formation at 0 K
Δ_f_H° (kJ mol^-1^)	Enthalpy of formation at 298.15 K and 1 bar
Δ_f_G° (kJ mol^-1^)	Gibbs energy of formation
H°-H_0_° (kJ mol^-1^)	Enthalpy difference
S° (J mol^-1^ K^-1^)	Molar entropy
C_p_ (J mol^-1^ K^-1^)	Heat capacity at constant pressure

The break between the tables consist of two rows. The first row contains the table number followed by the element symbol. The second row contains the full name of the element and the year it was prepared.

## 4 Impact

The NBS Tables of Chemical Thermodynamic Properties [1] is one of the most highly cited works in the history of NBS/NIST and is still used and cited in the academic, scientific, and engineering areas. In compliance with the Open Government Data Act [3], which "requires open government data assets to be published as machine-readable data," the NBS Tables are now accessible through CSV and Excel formats at https://doi.org/10.18434/M32124.
